# The left ventricle undergoes biomechanical and gene expression changes in response to increased right ventricular pressure overload

**DOI:** 10.14814/phy2.14347

**Published:** 2020-05-05

**Authors:** Vitaly O. Kheyfets, Melanie J. Dufva, Mario Boehm, Xuefeit Tian, Xulei Qin, Jennifer E. Tabakh, Uyen Truong, Dunbar Ivy, Edda Spiekerkoetter

**Affiliations:** ^1^ University of Colorado Denver Anschutz Medical Campus Aurora CO USA; ^2^ Department of Pediatrics Section of Cardiology Children’s Hospital Colorado Aurora CO USA; ^3^ Department of Medicine Division of Pulmonary and Critical Care Medicine Stanford University Stanford CA USA; ^4^ Vera Moulton Wall Center for Pulmonary Vascular Disease Stanford University Stanford CA USA; ^5^ German Center for Lung Research (DZL) Giessen Germany; ^6^ Cardiovascular Institute Stanford University Stanford CA USA; ^7^ Department of Pediatrics ‐ Division of Cardiology Virginia Commonwealth University Richmond VA USA

**Keywords:** interventricular coupling, left ventricle, pulmonary hypertension, right ventricle

## Abstract

Pulmonary hypertension (PH) results in right ventricular (RV) pressure overload and eventual failure. Current research efforts have focused on the RV while overlooking the left ventricle (LV), which is responsible for mechanically assisting the RV during contraction. The objective of this study is to evaluate the biomechanical and gene expression changes occurring in the LV due to RV pressure overload in a mouse model. Nine male mice were divided into two groups: (a) pulmonary arterial banding (PAB, *N* = 4) and (b) sham surgery (Sham, *N* = 5). Tagged and steady‐state free precision cardiac MRI was performed on each mouse at 1, 4, and 7 weeks after surgery. At/week7, the mice were euthanized following right/left heart catheterization with RV/LV tissue harvested for histology and gene expression (using RT‐PCR) studies. Compared to Sham mice, the PAB group revealed a significantly decreased LV and RV ejection fraction, and LV maximum torsion and torsion rate, within the first week after banding. In the PAB group, there was also a slight but significant increase in LV perivascular fibrosis, which suggests elevated myocardial stress. LV fibrosis was also accompanied with changes in gene expression in the hypertensive group, which was correlated with LV contractile mechanics. In fact, principal component (PC) analysis of LV gene expression effectively separated Sham and PAB mice along PC2. Changes in LV contractile mechanics were also significantly correlated with unfavorable changes in RV contractile mechanics, but a direct causal relationship was not established. In conclusion, a purely biomechanical insult of RV pressure overload resulted in biomechanical and transcriptional changes in both the RV and LV. Given that the RV relies on the LV for contractile energy assistance, considering the LV could provide prognostic and therapeutic targets for treating RV failure in PH.

## Graphical abstract

Right ventricular (RV) failure is a common endpoint in pulmonary hypertension. While most clinical and research efforts are focused on the RV, our research shows that the left ventricle undergoes bio‐mechanical and gene‐expression changes in response to RV pressure overload.

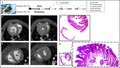

## 1 INTRODUCTION

Pulmonary hypertension (PH) is a progressive pulmonary vascular disease that ultimately leads to right ventricular (RV) functional decline and failure (Naeije & Manes, 2014). Chronic pressure overload in the RV‐pulmonary artery (PA) circuit initiates RV remodeling, which is initially adaptive and preserves the vascular‐ventricular coupling ratio (Kass & Kelly, 1992; Truong et al., 2015) (VVCR) by increasing contractility in response to increasing PA impedance. The adaptive remodeling process is characterized by RV changes in mechanics as well as structure (e.g. myocollagen fiber orientation) (Hill et al., 2014), fibrosis (Andersen, Nielsen‐Kudsk, Vonk Noordegraaf, & de Man, 2019), vascular changes, and metabolic dysfunction (Harvey & Chan, 2017). Ultimately though, when the RV contractility is no longer able to accommodate an increase in afterload, the remodeling is considered to become maladaptive (Sanz et al., 2012; Vanderpool et al., 2015) and is a precursor for bad outcomes resulting in RV failure and an increase in mortality (Vanderpool et al., 2015).

This perspective pathway of RV failure – that considers the RV as a pump in isolation from the left ventricle‐ has been the central focus for research investigating and targeting multiple aspects of RV functional decline in PH (Chin, Kim, & Rubin, 2005; Spiekerkoetter et al., 2017; Westerhof, Saouti, van der Laarse, Westerhof, & Vonk Noordegraaf, 2017). While some results have been promising, none are able to adequately restore RV pumping function to halt or reverse the progression to failure. We propose that this is because current research fails to consider the important contribution of the LV to RV systolic function (Damiano, La Follette, Cox, Lowe, & Santamore, 1991; Feneley et al., 1985), which could improve prognostic algorithms and reveal novel therapeutic targets. Damiano et al*.* showed that up to 75% of the pressure generated in the RV of dog hearts can be attributed to contractile energy transfer from the LV (Damiano et al., 1991). This is concerning, given that our previous studies have shown that LV torsion and torsion‐rate is significantly decreased in children with PAH, and is correlated with a decrease in RV contractility (Dufva et al., 2018). Other previous studies by our group in children with pulmonary arterial hypertension (PAH) also suggested that strain activated blood biomarkers might have originated from both ventricles (Kheyfets et al., 2015). Furthermore, patient‐specific simulations revealed that the LV myocardium is under elevated mechanical stress during RV pressure overload, and that RV structural remodeling could be the mechanism for decreased LV torsion rate that was observed in children with PAH (Kheyfets, Truong, Ivy, & Shandas, 2019).

However, because clinical data is etiologically heterogenous and does not offer tissue level biochemical analysis, we are presenting a rodent study that offers two important advantages: (a) PA banding (PAB) in a mouse study allows us to evaluate biomechanical and biochemical changes in the LV that occur solely as a result of a biomechanical insult in the RV‐PA axis; and (b) rodent studies allowed us to perform tissue level gene expression analysis. To this end, the overall objective of this work is to characterize LV biomechanical and biochemical changes that occur in response to biomechanically induced RV pressure overload.

## 2 MATERIALS AND METHODS

### 2.1 Study approval

All animal related experiments were carried out according to the guidelines outlined by the National Research Council (*Guide for Care and Use of Laboratory Animals*) and approved by the local Administrative Panel on Laboratory Animal Care (Stanford University, Protocol #27626).

### 2.2 Study protocol

**Figure 1:**
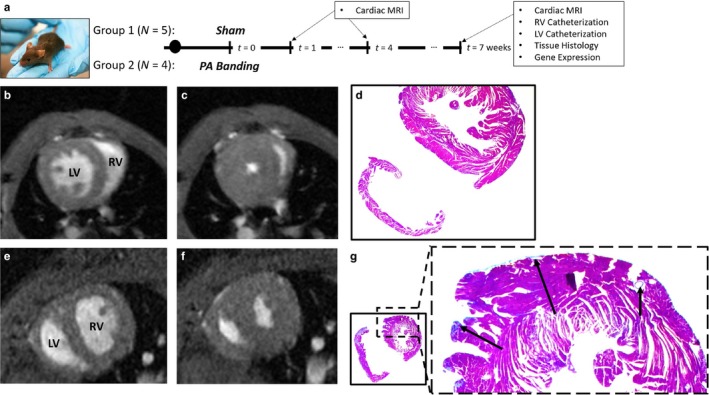
(a) Overall study design, timeline, and analysis; (b and c) Short axis cardiac MRI of a Sham mouse at (b) end‐diastole and (c) end‐systole. (d) Histology with collagen staining shown in blue for a randomly selected Sham mouse. (e, f) Short axis cardiac MRI of a PAB mouse at (e) end‐diastole and (f) end‐systole, respectively. (g) Histology with collagen staining in the LV of a randomly selected PAB mouse. Arrows show visible regions of fibrosis

Figure 1a shows an overview of the study design. Briefly, all male C57Bl6 mice (10–14 weeks of age) were randomly divided into two groups: (a) PAB and (b) sham surgery. In the 1st and 4th week after PAB, mice from all groups received a cardiac MRI with and without tissue tagging. On the 7th week, all mice underwent a final cardiac MRI, terminal intra‐cardiac hemodynamic catheterization (Spiekerkoetter et al., 2013), and were sacrificed to perform RV and LV tissue histology and measure gene expression.

#### 2.2.1 Pulmonary Arterial Banding (PAB) Mouse Model

PAB surgery was performed by suturing the around a 26G needle to induce an acute increase in PA impedance (Urashima et al., 2008). All mice subcutaneously received 0.05–0.1 mg/kgml Buprenorphine pre‐emptively along with constant Isoflurane (2%–3%) anesthesia before and during surgery, respectively. Only those animals that revealed 1‐week postsurgery peak pressure gradients – across the banded PA – greater than 20 mmHg were included in the study as part of the PAB group (measured by echocardiography, GE Vivid 7).

#### 2.2.2 Histomorphology

RV and LV myocardial tissues were formalin‐fixed, dehydrated, paraffin‐embedded, sectioned (3µm), and stained by Masson's trichrome stain as described in (Reddy et al., 2013). Area fraction analysis, using ImageJ, was utilized to measure the degree of myocardial fibrosis from Masson's trichrome stained sections.

#### 2.2.3 Gene expression measurements

Expression of selected miRNAs, which were chosen based on relevance in PH literature (Nishi et al., 2011; Reddy et al., 2012) and our previous studies on circulating miRNAs in children (V. O. Kheyfets et al., 2017), was measured using TaqMan probes and reverse transcriptase‐polymerase chain reaction (RT‐PCR). Below is a list of genes measured in LV tissue: (1) miR‐130; (2) miR‐93; (3) lethal‐7b (let‐7b); (4) miR‐34a; (5) α‐myosin heavy chain (MHC) mRNA; (6) β‐MHC mRNA; and (7) ATPase sarcoplasmic/endoplasmic reticulum Ca2 + transporting 2 (ATP2A2). All of these genes, except for miR‐34a and ATP2A2, were also measured in the RV.

The relative expression of reported genes was determined using the ΔΔCt method (Livak & Schmittgen, 2001). GAPDH and miRSN202 were used as the reference genes for RNA and miRNA expression, respectively. All values reported were normalized the mean of the Sham cohort.

#### 2.2.4 MRI measurements

All cardiac MRIs were performed, under anesthesia, on a 7T Bruker BioSpec scanner (Bruker Co., MA, USA) at 3 time points: 1‐week after PAB; 3‐weeks after PAB; and 7‐weeks after PAB just before terminal catheterization. LV torsion rate was measured from tagged MR images using a post‐processing protocol described previously in (Dufva et al., 2018).

#### 2.2.5 RV and LV function

Ventricular function and mechanics in all animals within the study was assessed by 4 metrics: (1) maximum LV torsion during systole; (2) LV torsion rate; (3) LV and RV ejection fraction (*EF*), and (4) stroke work (*W*). EF was measured by semi‐automatic segmentation of the ventricular cavity at end‐systole and end‐diastole by MRI. Finally, *W* ‐a measure of the stroke work required to displace the blood volume of a single heart beat ‐ was measured as the area bounded by the pressure‐volume loop (Suga, 1979), where pressure was measured by terminal catheterization within 1‐day of MRI (used for the volume measurement).

#### 2.2.6 Data analysis

Important Note: For various reasons, some measurements were occasionally unavailable for a specific mouse. For example, right heart catheterization was completed for one of the Sham mice, but left heart catheterization was not possible due to complications during surgery. Therefore, all presented results include the sample size (N) used to generate that plot. The authors declare that a mouse was never excluded from a specific analysis to adjust results, but only due to data availability.

All statistical analysis, waveform signal processing, and calculation presented in this manuscript was coded using Matlab (Mathwors). Means within a longitudinal test were compared using a paired *t*‐test. All other pairwise comparisons were made using the nonparametric two‐sided Wilcox rank sum test. For datasets with a lot of zeros (e.g. level of fibrosis was zero in many of the Sham animals), the proportions of non‐zeros within the two groups was assessed using the Chai‐square test (*p* < .05 considered significant).

The relationship between variables (e.g. gene expression vs. ventricular torsion rate) was evaluated using coefficient of determination (R^2^), with occasional logarithmic transformation to correct for skewed distributions.

*Pressure and Volume Waveform Analysis –* RV and LV pressure waveforms were collected for 3 Sham and 4 PAB mice at a sampling frequency of 2000 Hz. A typical waveform contained 3 to 4 cardiac cycles, where each cycle was extracted using the periodogram power spectral density estimate and the mean cycle was recovered by Fourier transforms with 10 harmonics.

Ventricular volume waveforms were reconstructed from 20 images within the duration of the cardiac cycle (using CVI42). The waveform was reconstructed using Fourier Series with 3 harmonics.

To construct pressure‐volume curves, the pressure (P) and volume waveforms were synchronized at end‐diastole. While all MRI images begin at end‐diastole, in the pressure waveform, this point was identified as the second peak of $\partial^{2}P/\partial t^{2}$.

## 3 RESULTS

### 3.1 Structural changes in RV and LV mechanics after PAB

Figure 1b‐g shows an example MRI and tissue histology comparing Sham and PAB mice. Qualitative MRI analysis (Figure 1b,c,e,f) reveals notable septal flattening and RV free wall thickening after PAB. RV and LV tissue histology shows ‐as expected‐ significantly increased fibrosis in the RV at week 7 after PAB, relative to the LV (15.0 ± 6.22% in RV vs. 1.12 ± 0.47% in the LV, *p*_2‐tailed_ < .05). However, all 4 PAB mice revealed some nonzero level of fibrosis in the LV as well. In contrast, only 1 out of the 5 Sham mice had 0.23% fibrosis in the LV, which is below 1 standard deviation of the fibrosis amount in the PAB sample (Chi‐square test with a single Sham mouse positive for fibrosis reveals a *p* = .016, but a Yates continuity correction makes the relationship insignificant). Qualitatively analyzing example histology with collagen staining in a single Sham (see Figure 1d) and PAH (see Figure 1g) mouse shows collagen in the interventricular septum and perivascular fibrosis in the LV free wall of PAB mice.

### 3.2 Functional and gene expression changes in the RV after PAB

**Figure 2:**
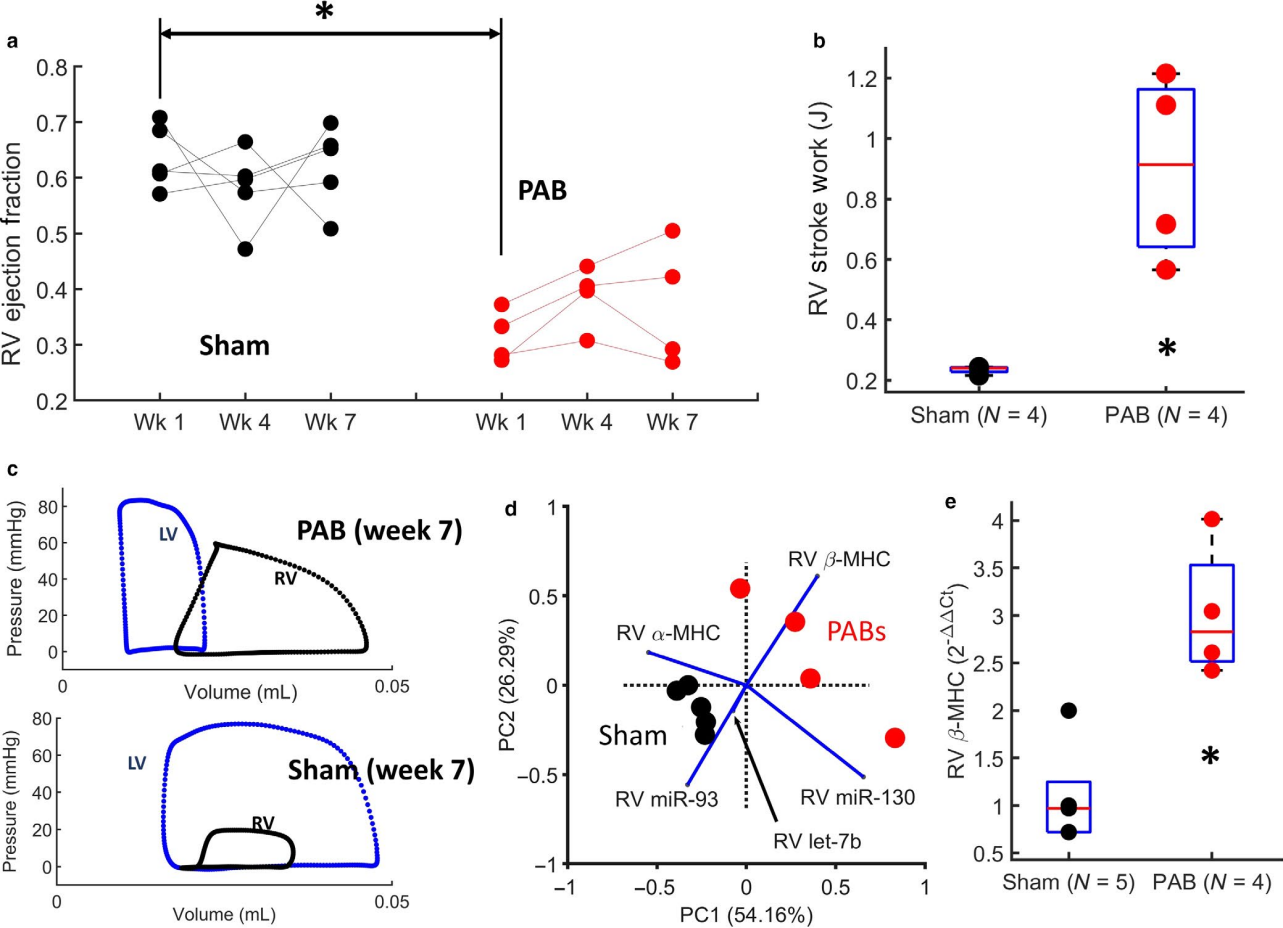
Pulmonary arterial banding results in significant changes to RV systolic function and gene expression. (a) Slope graphs showing longitudinal changes in RV ejection fraction for PAB and Sham mice. The plot shows a significant decrease in RV EF 1‐week after PAB. (b) RV stroke is significantly increased in mice after 7‐weeks of PAB, relative to the Sham animals. (c) Example ventricular pressure‐volume loops in Sham and PAB mice use to compute ventricular stroke work. (d) Principal component analysis biplot of the first 2 principal components, showing Sham and PAB animals cluster based on 5 genes. (e) RV myocardium shows significantly increased β‐MHC expression at 7‐weeks after PAB. In the box plots shown, the central mark indicates the mean, with bottom and top edges showing the 25th and 75th percentiles, respectively. The symbol: * indicates statistical significance of the Wilcox rand sum test (*p*_2‐tailed_ < .05)

Figure 2 compares RV function and gene expression between Sham and PAB mice. The RV shows significantly decreased EF 1‐week after PAB surgery (Figure 2a), which was maintained for the duration of the experiment (to week 7). However, relative to the Sham mice, RV stroke work (measured as the area within the pressure‐volume loops – see Figure 2c) in PAB mice was increased by over 300% (RVSW = 0.23 ± 0.02 J in Sham vs. 0.90 ± 0.31 J in PAB, *p*_2‐tailed_ < .05; see Figure 2b). Comparing RV gene expression between the two groups, miR‐93 (miR‐93 2^−ΔΔCt^ = 1.01 ± 0.18 in Sham vs. 0.42 ± 0.16 in PAB, *p*_2‐tailed_ < .05) and β‐MHC (see Figure 2e) were significantly elevated (*p*_2‐tailed_ < .05) in PAB animals, relative to Sham. Performing PCA (after natural log transformation of the raw data) on all 5 measured genes (miR‐93, let‐7b, miR‐130, and the genes coding for α & β‐MHCs) clustered PAB and Sham animals within the plane of the first two principal components (describing 80.45% of the variance in the data – see Figure 2d).

### 3.3 Changes in LV systolic function, contractile mechanics, and stroke work

**Figure 3:**
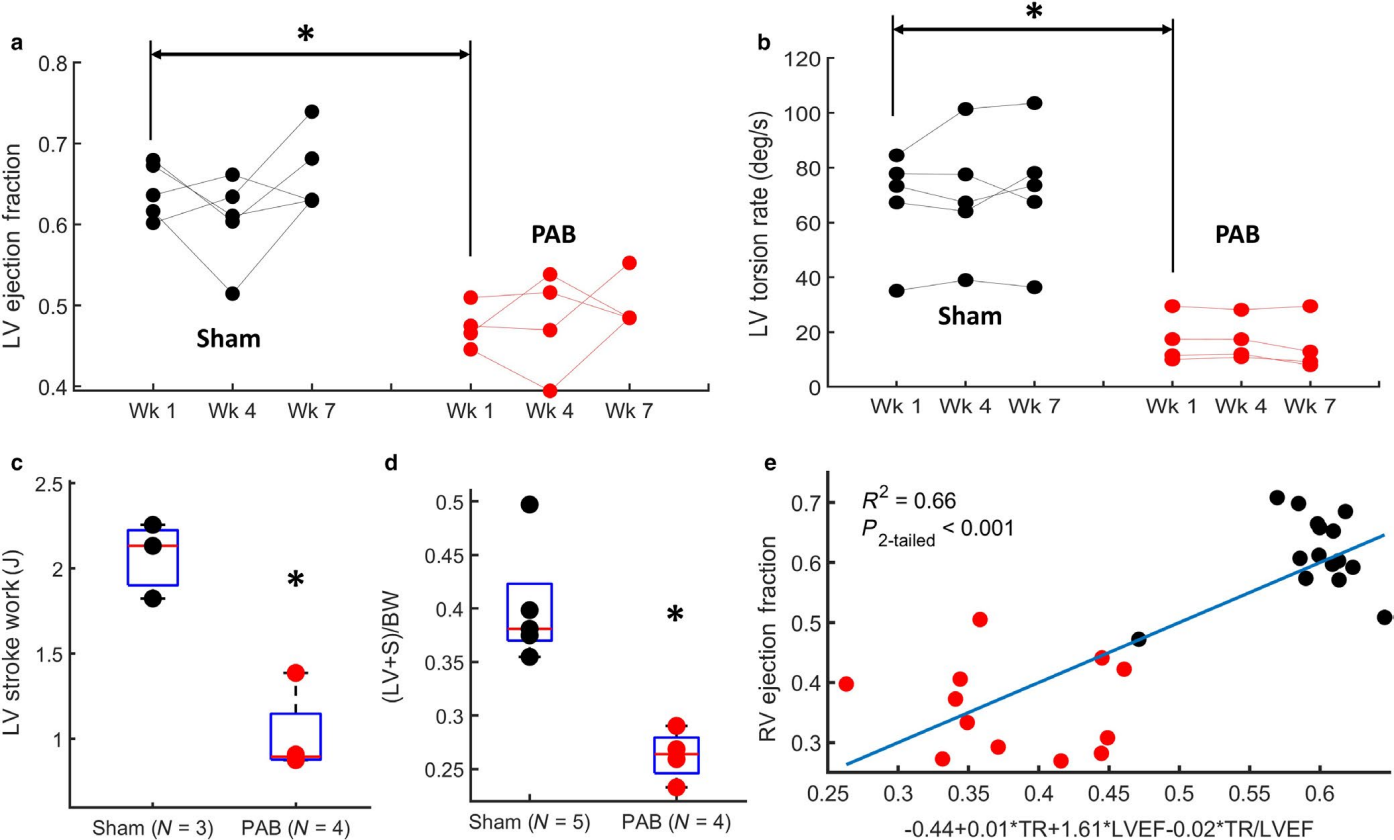
An acute pressure‐overload insult to the RV leads to biomechanical and functional changes in the LV. (a, b) LV ejection fraction and torsion rate compared longitudinally using slope graphs and between control and PAB mice. LV ejection fraction (LVEF) is significantly decreased 1‐week after PAB and remains decreased for the duration of the experiment. (b) LV torsion rate (TR) during systole is significantly decreased 1‐week after PAB and remains decreased for the duration of the experiment. (c) LV stroke work is significantly decreased at week 7 after PAB, relative to Sham mice. (d) LV and septal weight normalized by mouse body weight ((LV + S)/BW)) is significantly decreased in mice at 7‐weeks after PAB. (e) Multivariate linear regression model between declining LV systolic function/mechanics ‐measured by maximum LV torsion rate and LV ejection fraction ‐ and declining RV ejection fraction. Red and black dots represent PAB and Sham animals, respectively. In the box plots shown, the central mark indicates the mean, with bottom and top edges showing the 25th and 75th percentiles, respectively. The symbol: * indicates statistical significance of the Wilcox rand sum test (*p*_2‐tailed_ < .05)

Similar to the RV, LV EF and torsion‐rate were analyzed at 1, 4, and 7 weeks after PAB surgery. PAB mice revealed a significant decrease in both LV EF (*p*_2‐tailed_ < .05) and torsion rate (*p*_2‐tailed_ < .05) at 1‐week after surgery, relative to controls, without significant changes in either metric for the duration of the experiment. Unlike in the RV, LV stroke work was reduced by half after PAB (LVSW = 2.07 ± 0.22 J in Sham vs. 1.01 ± 0.25 J in PAB, *p*_2‐tailed_ < .05). In fact, as opposed to Sham animals where LV work is 9 times greater than RV work, the stroke work is roughly equal in the two ventricles after PAB. Furthermore, the weight of the LV and septum, relative to the total body weight of the animal, is also significantly decreased after PAB (see Figure 3d).

Figure 3e also shows an association between combined declining LV ejection fraction and LV torsion rate into a multivariate model, versus declining RV ejection fraction (*p*_2‐tailed_ < .001). This plot combines all temporal measurements of PAB and Sham animals. Therefore, since the study protocol performed MRI measurements in 4 PAB animals and 5 Sham animals, at 3 time points, there are a total of 12 red dots (representing PAB animals) and 15 black dots (representing Sham animals). Similar associations were found between maximum LV torsion rate and RV ejection fraction (*R* ^2^ = .50, *p*_2‐tailed_ < .001).

### 3.4 Changes in gene expression in the LV – and the relationship to LV function

We measured LV tissue expression of 4 miRNA candidates (miR‐34a, miR‐130, miR‐93, and let‐7b) that were chosen based on previous data and a literature review, ATP2A2 gene to evaluate calcium dynamics, and two mRNAs coding for contractile proteins (α‐MHC, β‐MHC). Gene expression in the LV was compared between PAB and Sham mice, and ‐within the PAB cohort‐ correlated with LV contractile mechanics (maximum LV torsion and LV torsion rate).

**Figure 4:**
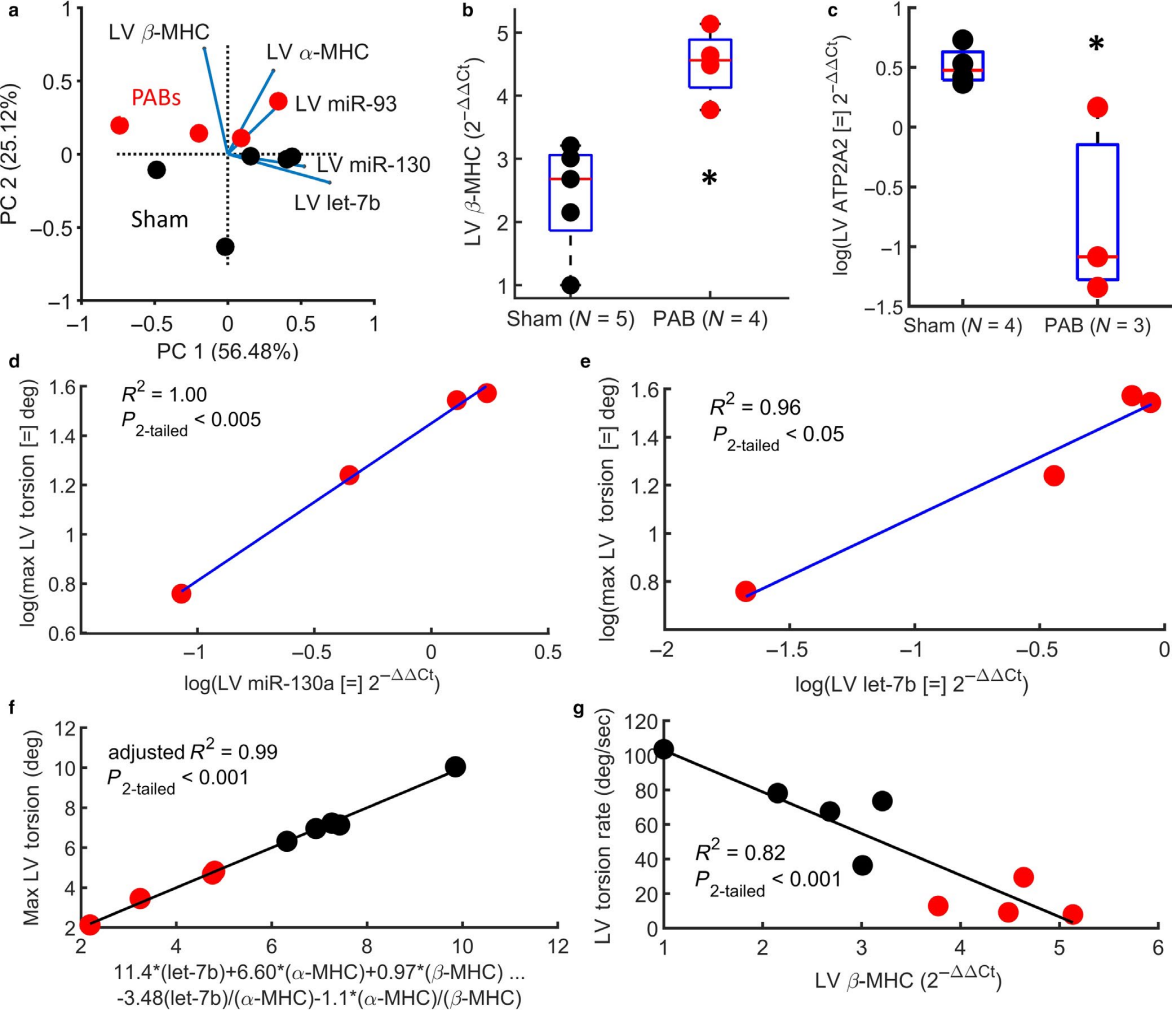
Gene expression is altered in the LV of mice after PAB surgery, and changes in expression are correlated to changes in contractile mechanics. (a) Principal component biplot shows that LV gene and protein expression in the LV stratify PAB and Sham mice along PC2. (b, c) β‐MHC expression in the LV is significantly increased after PAB surgery and ATP2A2 ‐which governs myocardial calcium dynamics‐ in the LV is significantly decreased. (d, e) Downregulation of miR‐130a and let‐7b – respectively‐ in the LV are correlated with a decrease in maximum LV torsion during systole. (f) Stepwise linear regression with interaction reveals a multivariate model of gene expression (including genes coding for contractile proteins) in the LV described 99% of the variability in maximum LV torsion. (g) An increase in β‐MHC expression in the LV is correlated with a decrease in LV torsion rate. In the box plots shown, the central mark indicates the mean, with bottom and top edges showing the 25th and 75th percentiles, respectively. The symbol: * indicates statistical significance of the Wilcox rand sum test (*p*_2‐tailed_ < .05)

Principal component analysis (PCA) was used for dimensionality reduction (after natural log transformation of the raw data) of all biochemical analysis performed for the LV with the same available sample size. This included miR‐130, miR‐93, let‐7b, α‐MHC, and β‐MHC. Figure 4a shows a biplot of the first two principal components, which explain 81.58% of the variance within the data and stratify the PAB and Sham groups along PC2. When comparing mean gene expression between Sham and PAB groups, β‐MHC expression was significantly upregulated in the PAB cohort (β‐MHC 2^−ΔΔCt^ = 2.41 ± 0.88 in Sham vs. 4.51 ± 0.56 in PAB, *p*_2‐tailed_ < .05) (see Figure 4b), which has major implications for function and contractile mechanics (see *Discussion*). Also, the ATP2A2 gene – measured in 3 PAB and 4 Sham mice‐ was significantly downregulated in the LV of PAB mice (*p*_2‐tailed_ < .05), relative to the sham cohort (see Figure 4c). Mir‐34a was not detected in the LV and was therefore omitted from all analysis.

When comparing gene expression in the LV against contractile mechanics, we observed that a decrease in maximum LV torsion during systole is correlated with decreasing miR‐130 (*R* ^2^ = 1.00, *p*_2‐tailed_ < .05) and let‐7b (*R* ^2^ = .96, *p*_2‐tailed_ < .05) in the PAB cohort (Figure 4d,e), but the small available sample size makes it difficult to draw any meaningful conclusions. When combining Sham and PAB mice into a stepwise linear regression (with interactions), let‐7b, α‐MHC, and β‐MHC are revealed as the significant variables in the multivaraite model (miR‐130 and miR‐93 were not significant) that explaines 99% of the variability in maximum LV torsion during systole (see Figure 4f). Finally, β‐MHC expression in the LV was significantly correlated with LV torsion rate in both Sham and PAB mice, suggesting the importance of the β isoform ‐within the myosin contractile apparatus‐ for organ level kintetic energy generation.

## 4 DISCUSSION

The data presented in this manuscript shows that the mouse LV undergoes biomechanical, functional, and gene expression changes in response to RV pressure overload. The overall application of this work is targeted towards studying pulmonary hypertension, where research and clinical focus has primarily been focused on the RV (Chin et al., 2005; Spiekerkoetter et al., 2017; Westerhof et al., 2017).

There is an undoubtable mechanical energy transfer occurring between the LV and RV. In addition to sharing a septum and being physically connected at the inter‐ventricular sulcus, multiple layers of muscle fibers are interlaced across both ventricles and the entire heart is enclosed in a shared pericardium (Barnard & Alpert, 1987; Haddad, Hunt, Rosenthal, & Murphy, 2008). Each ventricle must meet the circulatory needs of the adjacent vascular bed, with a universal property of stress and contractile force sharing (Pasque & Wechsler, 1986). In systole, RV contraction slightly precedes that of the LV, but the maximum *d(Pressure)/d(time)* occurs simultaneously in normal sinus rhythm (Feneley et al., 1985). Contractile kinematics are drastically different between the two ventricles. The LV contracts by radial constriction and longitudinal shortening (Sorger, Wyman, Faris, Hunter, & McVeigh, 2003). The act of LV contraction pulls the inter‐ventricular septum to the left, causing a bellows‐like flattening of the RV free wall and contributing to 20%–70% of the contractile pressure generation (Damiano et al., 1991; Santamore & Dell'Italia, 1998). In fact, a purely passive RV free wall is shown to have little impact on contractile pressure generation in the RV‐PA axis (Hoffman, Sisto, Frater, & Nikolic, 1994).

Therefore, thoroughly characterizing biomechanical and biochemical changes in the LV ‐that occur in response to RV pressure overload in PH‐ can serve as:

1. ***A prognostic marker of inter‐ventricular mechanical energy transferred to the RV.*** Our previous modeling studies have shown that a passive or stiff RV can dramatically change LV contractile mechanics and LV myocardial stress (Kheyfets et al., 2019). Therefore, the LV can serve as a reliable marker of structural changes in the RV.
2. ***A therapeutic target to improve RV function by targeting LV mechanical energy assistance.*** Damiano *et al.* (Damiano et al., 1991) showed that LV contraction ‐in a healthy dog heart‐ is responsible for more than 75% of the pressure generation in the RV. However, it is unclear how RV structural changes, mechanical changes, or changes to inter‐ventricular septum morphology – a common phenotype of RV pressure overload (Haddad et al., 2008)‐ impact this energy transfer.

### 4.1 LV contractile mechanics and systolic function after PAB

Based on our results, LV torsion rate and RV/LV EF are decreased within a week of PAB, but then remain in this –plateaued‐ decreased state for the duration of the experiment (to week 7). This is consistent with multiple studies that showed cardiac growth and remodeling in the RV occurs within the first week of the initial insult (Hill et al., 2014; Ivey et al., 2018). Ivey et al. (2018) showed that fibroblast proliferation peaks within the first week after injury and remains high for at least the following week. Hill et al. (2014) also showed that myofiber and collagen re‐orientation, and hypertrophy, in the RV free‐wall occur within the first week after PAB. Therefore, these findings would suggest that the observed changes in LV contractile mechanics, which also occur within the first week of banding, are possibly due to the mechanical burden from structural changes within the RV. This interpretation is reinforced by our previous work, where we developed a computational model of the bi‐ventricular heart (Kheyfets et al., 2019) and simulated findings from Hill *et al.’s* study to show that acute changes in RV structure caused an acute decrease in LV torsion.

Consistent with a previous study of rats at 6‐weeks after PAB (Faber et al., 2006) and a piglet PH model (Guihaire et al., 2015), RV stroke work and contractility is increased in response to the resulting pressure‐overload. However, in contrast with (Faber et al., 2006) (where no significant difference in LV stroke work was reported after PAB) we found a significant decrease in LV stroke work at 7‐weeks after PAB. Sugen5416 + Hypoxia rat models of PAH support our finding and also revealed decreased LV stroke work, relative to control rats (Rawat et al., 2014), along with LV diastolic dysfunction.

Ventricular stroke work was calculated as the area within the pressure‐volume curve, which can be interpreted as the amount of work done by the myocardium to displace a stroke volume of blood against a given afterload (“external work” (Timmer et al., 2010)). Our study showed that – under normal conditions‐ LV stroke work is much higher than in the RV. However, after PAB, the RV stroke work must increase to overcome the increased afterload, and LV stroke work decreases to almost RV levels. We postulate that if the total work of LV contraction (see Equation 1) is assumed to have stayed constant before and after PAB, it is likely that more work is required to overcome the burden of the RV after banding, so less work is being allocated for LV blood displacement. Although, systemic cardiac output was not measured to confirm this.

(1) $$\begin{array}{cc} \text{Total Work of LV Contraction}\left(\text{Watts}\right) & =\text{Work done to displace blood to the systemic circulation}\\ & +\text{Work done to ovecome bourden of RV}\\ & +\text{Work transfered to the RV}. \end{array}$$

### 4.2 Structural changes in the LV in response to RV pressure overload

Consistent with previous reports (Aguero et al., 2014; van de Veerdonk, Bogaard, & Voelkel, 2016), we observed severe interstitial and perivascular fibrosis in the RV free wall after PAB. When looking in the LV, the septum and the inter‐ventricular sulcus also revealed slight but significantly elevated fibrosis in PAB animals, relative to sham animals, with some perivascular fibrosis even visible in the LV free wall, but to a much lesser extent than in the RV. This is supported by MRI studies in patients with pulmonary hypertension that have shown elevated T1 times in the entire LV, which is correlated with changes in the eccentricity index (Reiter et al., 2017). The aforementioned computational studies in our lab have suggested a potential mechanism by showing that increased RV pressure overload causes increased LV myocardial stress (Kheyfets et al., 2019), which is a biomechanical initiator for fibroblast activation (Espeland, Lunde, B, Gullestad, & Aakhus, 2018). However, these modeling studies were conducted on a human heart model and an actual estimate of myocardial stress was not available for the mice in this study. Furthermore, while evidence of LV perivascular fibrosis may be a marker of elevated myocardial stress, there has been debate about its implication for function. Traditional thinking has suggested that myocardial fibrosis depresses electrical and mechanical function in the heart (Espeland et al.., 2018), but some studies have suggested that fibrosis is both an adaptive and maladaptive response (Andersen et al., 2019). Therefore, it is unlikely that the minimal fibrosis we observed in the LV of PAB mice is somehow contributing to reduced torsion rate or EF, but is likely a benign response to biomechanical and biochemical stimuli.

Ideally, we were looking to correlate gene expression and fibrosis in the LV with an estimate of LV myocardial stress. Previous studies have derived estimates of LV myocardial stress based on a force‐balance between LV cavity pressure and the balancing stress of the myocardium (Arts, Bovendeerd, Prinzen, & Reneman, 1991; Denslow, Balaji, & Hewett, 1999). These equations are derived without any regard for the septal geometry or the influence of the RV. Therefore, this estimate is not applicable for this study, which proposes that the elevated LV stress is a result of RV remodeling and pressure overload. Thus, ongoing studies are attempting to estimate LV stress from finite element analysis and will be reported in future papers.

### 4.3 Gene expression changes in the LV during RV pressure overload

Due to budget constraints, we were limited in the number of genes that could be evaluated for this preliminary assessment of LV biochemical changes in response to a RV‐PA mechanical injury. We measured α and β myosin heavy chain (MHC) mRNA expression because they were known to be miss‐regulated in the RV after PAB (Bartelds et al., 2011) and implicated in myocyte contractile depression in the LV (Dorn, Robbins, Ball, & Walsh, 1994) (with upregulation of β‐MHC). Therefore, we suspected that upregulated β‐MHC would be correlated with changes in LV myocardial contractile speed, which would tie a biochemical response to an already observed biomechanical phenomena in the LV of patients with increased RV‐PA impedance. In addition to changes in genes that code for contractile proteins, we also chose to measure changes in ATP2A2 gene expression due to the important role of calcium dynamics in normal cardiac function (Vangheluwe, Sipido, Raeymaekers, & Wuytack, 2006). Finally, for the remaining markers, we chose to focus on miR expression instead of specific genes to expose a broader range of pathways for future analysis. To our knowledge, we are the first to relate gene expression changes in the LV to specific markers of contractile mechanics during chronic RV pressure overload. However, the associations found in our data is purely observational and does not suggest any mechanistic link. We chose 4 miR candidates (miR‐93, miR‐130a, miR‐34a, let‐7b), based on their expected physiological relevance from literature and our unpublished preliminary circulating miRNA data in children with PH (Note: we have published circulating miR candidates associated with vascular dysfunction (Kheyfets et al., 2017), but have not published circulating miR candidates associated with ventricular dysfunction). Although, there are additional possible miRNA targets in the LV that have been revealed in Sugen + Hypoxia rat models (e.g. miR‐31a, miR‐208b) (Joshi et al., 2016), which we plan to explore in future microarray studies.

Previous studies have reported that miR‐93 is upregulated in the RV after PAB (Batkai, Bar, & Thum, 2017; Thum & Batkai, 2014), but is downregulated in the LV after aortic constriction (Batkai et al., 2017). However, our study found that miR‐93 was downregulated in the RV after PAB, and was unchanged in the LV. Therefore, future studies will need to determine if miR‐93 regulation is a ventricle‐specific response, or if the degree and timing of mechanical stimuli in the RV and LV by PAB is the key to triggering specific changes in miR‐93 expression.

As anecdotal evidence of the intrinsic coupling between biomechanical and biochemical changes observed in the LV after PAB, dimensionality reduction of miR and MHC mRNA expression in the LV reveals PAB and Sham group clustering along the second principal component. Within the PC1 and PC2 plane, vectors corresponding to each gene show that α‐MHC, β‐MHC, and miR‐93 have the largest impact on where a data point will lie along PC2. This is consistent with previous studies, as we will outline in the remainder of this discussion.

Principal component analysis did not include miR‐34a because of differences in sample size, but miR‐34a was not differentially expressed in either ventricle after PAB, which is inconsistent with previous studies that found an upregulation in the RV under comparable experimental conditions (Thum & Batkai, 2014).

Given that our primary interest is focused on LV contractile mechanics under elevated RV pressure, we investigated how gene expression was associated with maximum LV torsion and torsion rate. A decrease in miR‐130a and let‐7b expression in the LV was linearly correlated with a decrease in maximum LV torsion. However, the expression of miR‐130a or let‐7b were not significantly decreased in the LV of PAB mice, relative to the Sham mice. In previous studies, upregulation of miR‐130a in cardiac fibroblasts promoted a profibrotic phenotype (Li, Bounds, Chatterjee, & Gupta, 2017), but we found no correlation to LV fibrosis in our study and the resulting association with LV torsion appears to be in the opposite direction. However, the small sample size used in our study makes it more suitable to warrant additional research, rather than drawing absolute conclusions in contradiction to previous research.

The let‐7 miR family is highly expressed in the cardiovascular system, but is generally over‐expressed in cardiovascular disease (Bao et al., 2013; Divakaran & Mann, 2008). However, some studies have shown a downregulation of RV let‐7b during RV heart failure in response to Sugen (SU5416)+Hypoxia but not after PAB (Thum & Batkai, 2014). A downregulation of circulating let‐7b is also associated with acute myocardial infraction (Long et al., 2012), and therapeutically targeting up‐regulation of the let‐7 family attenuated cardiac hypertrophy (Yang, Ago, Zhai, Abdellatif, & Sadoshima, 2011). Our data showed no statistical change between PAB and Sham group in the let‐7b expression within the RV or the LV. However, there was a significant correlation between down‐regulated let‐7b expression and maximum LV torsion during systole, but without any indication if this is the cause or effect of changing biomechanics. Previous studies from other fields have suggested that downregulation of members within the let‐7 family could be due to mechanosensitivity (Mohamed, Hajira, Lopez, & Boriek, 2015), which would be consistent with our findings, but this would need to be confirmed in the heart.

In a healthy human heart, 20%–40% of the total MHCs consist of α‐MHC mRNAs (Lowes et al., 1997; Nakao, Minobe, Roden, Bristow, & Leinwand, 1997; Sucharov et al., 2004), which can decrease by 75% in pulmonary hypertension (Lowes et al., 1997) and coupled to an upregulation in β‐MHC in both ventricles. This phenomenon is also seen in a rat PH model, which under healthy conditions expressed higher levels of α‐MHC when compared with humans (Nishi et al., 2011; Yen et al., 2010). To our knowledge, we are the first to show an increase in β‐MHC expression in the LV of RV pressure‐overloaded rodents, which is correlated with decreased LV torsion rate. While the α and β isoforms contain 93% similarity in amino acid identity, rat myocytes fragments expressing 12% α‐MHC generated 53% more contractile power than fragments expressing only β isoforms (Herron & McDonald, 2002). However, directly exposing a causal link between β‐MHC overexpression and LV rotational mechanics will have to be addressed in future studies.

Overall, for both PAB and Sham animals, a multivariate model of let‐7b, α‐MHC, and β‐MHC explains 99% of the variability in maximum LV torsion. This does not offer any causal explanation, but it does reveal an intricate relationship between myocardial biomechanics and contractile gene expression, which should be explored further.

### 4.4 LV calcium dynamics after PAB

The ATP2A2 gene codes for the sarco‐/endoplasmic reticulum calcium‐ATPase 2 (SERCA2a)‐encoding mRNA, which controls multiple aspects of cardiac muscle contractility and energy management through Ca^2+^ signaling (Kranias & Hajjar, 2012; Vangheluwe et al., 2006). Numerous studies have shown that SERCA2a expression in the LV is reduced during end‐stage ventricular failure that's initiated by pressure‐overloaded hypertrophy (Arai, Matsui, & Periasamy, 1994; Levitsky, de la Bastie, Schwartz, & Lompre, 1991), but it has never been explicitly related to myocardial mechanical stress. In our current study, there is no evidence of pressure‐overloading in the LV after PAB, given that LV systolic pressure is not increased in the PAB group relative to the Sham group, but ATP2A2 levels are decreased. As previously explained, simulation studies did show elevated LV myocardial stress (Kheyfets et al., 2019) under RV pressure‐overload in a reconstructed child's heart, but these simulations will be repeated in mouse‐specific hearts in future work.

Downregulation of SERCA2a in both the RV and pulmonary circulation is a well‐documented phenotype of PAH, and forced over‐expression decreases vascular proliferation, RV hypertrophy, and RV fibrosis to normal levels (Hadri et al., 2013). Aortic banding in rats has shown a decrease in SERCA2a expression and activity (Miyamoto et al., 2000), but, ‐to our knowledge‐ calcium dynamics in the LV, under RV pressure overload, has not been investigated. To this end, our data shows that strategies to increase SERCA2a expression could work to benefit both ventricles under RV pressure overload, which warrants further investigation.

### 4.5 Clinical and translational importance

Previous research from our group and elsewhere have documented biomechanical and structural changes (Dufva et al., 2018; Reiter et al., 2017) within the LV of patients with pressure overload in the RV‐PA axis. However, it was unclear if this was a result of biomechanical or biochemical signaling. The mice in our study were only mechanically stimulated through PAB, which does not rule out biochemical signaling, but confirms inter‐ventricular biomechanical signaling as a strong factor in LV contractile mechanics. This finding not only experimentally supports our previous computational modeling studies (Kheyfets et al., 2019), but also reveal LV changes in gene expression. Therefore, when considering the amount of contractile energy being transferred from the LV to assist in RV contraction, these changes in LV mechanics and transcriptomics offer potential prognostic and therapeutic targets.

### 4.6 Limitations

Both the Sham and PAB group of mice had a small sample size, which created a major limitation of this study. Furthermore, the sample size in some analyses could not use the entire cohort due to complications in data acquisition (e.g. catheterization was not possible in some animals). However, all relationships presented revealed statistical significance.

The expression of genes explored within the LV was limited. Financial constraints prevented us from performing microarray analysis, but the current results offer justification for additional gene expression studies in the LV.

The results presented in this study are limited to correlation, without addressing causality. Future studies will be prospectively designed to draw causal inference (Pearl, 2009) between RV mechanics, LV mechanics, and gene expression.

## 5 CONCLUSIONS

The clinical and translational significance of this study is the observation that purely biomechanical insults within the RV induced significant changes in LV mechanics, gene expression, and contractile protein expression. We found that LV ejection fraction, stroke work, and torsion rate were all significantly decreased at 7‐weeks after induced RV pressure overload by pulmonary arterial banding. Furthermore, we found changes within the LV in gene expression, calcium handling, and contractile protein expression, which were highly correlated to a decrease in maximum LV torsion and torsion rate.

These changes within the LV are currently overlooked in patients with RV pressure overload, but they could serve as important prognostic markers of RV function and outcome. Furthermore, given that the RV heavily relies on mechanical energy transfer from the LV, these findings could be used as a therapeutic target for mitigating or even reversing RV functional decline in PH.

## FUNDING AND DISCLOSURES

This work was supported by the National Institutes of Health: NIH K25 HL133481 and the Jayden DeLuca Foundation. MB was supported by a Max Kade Foundation postdoctoral fellowship. ES received support by NHLBI R01 HL128734, Department of Defense PR161256, Wall Center for Pulmonary Vascular Disease Stanford. DI has received research support from Actelion Pharmaceuticals Ltd, Eli Lilly & Co, and United Therapeutics; the University of Colorado contracts with Actelion Pharmaceuticals Ltd, Bayer HealthCare, Eli Lilly & Co, and United Therapeutics for DD Ivy to be a consultant. DD Ivy receives grant funding from the National Institutes of Health and the Food and Drug Administration.
